# Direct and Indirect Genetic Effects on Child ADHD Traits in Early and Mid-Childhood: Trio Genome-Wide Complex Trait Analyses in a Large Norwegian Birth Registry Cohort

**DOI:** 10.1007/s10519-026-10273-1

**Published:** 2026-06-29

**Authors:** Daniel L. Wechsler, Espen M. Eilertsen, Yasmin I. Ahmadzadeh, Rosa Cheesman, Nicoletta Adamo, Laurie J. Hannigan, Eivind Ystrom, Tom A. McAdams

**Affiliations:** 1 Social, Genetic and Developmental Psychiatry Centre, Institute of Psychiatry, Psychology and Neuroscience, King’s College London, London, UK; 2 Centre for Brain and Cognitive Development, Birkbeck, University of London, London, UK; 3 Department of Psychology, Institute of Psychiatry, Psychology & Neuroscience, King’s College London, London, UK; 4 PROMENTA, Department of Psychology, University of Oslo, Oslo, Norway; 5 PsychGen Centre for Genetic Epidemiology and Mental Health, Division of Public Health and Prevention, Norwegian Institute of Public Health, Oslo, Norway; 6 Department of Child and Adolescent Psychiatry, Institute of Psychiatry, Psychology & Neuroscience, King’s College London, London, UK; 7 Child and Adolescent Mental Health Services, South London and Maudsley NHS Foundation Trust, London, UK; 8 Psychiatric Genetic Epidemiology group, Research Department, Lovisenberg Diaconal Hospital, Oslo, Norway; 9 Population Health Sciences, Bristol Medical School, University of Bristol, Bristol, UK; 10 Department of Child Health and Development, Norwegian Institute of Public Health, Oslo, Norway; 11 Centre for Research on Equality in Education, University of Oslo, Oslo, Norway

**Keywords:** ADHD, Indirect genetic effects, Genetic nurture, Trio-GCTA, Parent-child, MoBa

## Abstract

Genetic variants in family members may exert environmentally mediated indirect genetic effects on children’s attention-deficit hyperactivity disorder (ADHD) traits. We set out to quantify the indirect genetic effects of parents’ genotypes on children’s ADHD traits as measured in early and mid-childhood. We analyzed data from genotyped trios of children, mothers, and fathers of European ancestry from the Norwegian Mother, Father and Child Cohort Study (MoBa) birth cohort. Child ADHD data were available for analytic subsamples of 12,374 trios at age 5 (children 50.5% male), and 12,714 trios at age 8 (children 50.9% male). We quantified direct genetic effects of children’s genotype and indirect effects of mothers’ and fathers’ genotypes on children’s ADHD traits at each age. Differing patterns of direct and indirect genetic effects were observed in early and mid-childhood, with predominantly maternal indirect genetic effects on ADHD traits at age 5, and roughly equal direct genetic effects and maternal indirect effects on ADHD traits at age 8. However, large standard errors hindered confident inference as to the relative importance of specific effects, and model fit comparisons ultimately favored models with no indirect genetic effects at age 5 and 8, as well as no direct genetic effects at age 5. We do not find clear evidence of indirect genetic effects of maternal or paternal genotype on child ADHD traits at ages 5 and 8, nor direct genetic effects on child ADHD traits at age 5. While previous findings of indirect genetic effects on ADHD subdomains at ages 3 and 8 broadly support the plausibility of indirect genetic effects operating on total ADHD scores at ages 5 and 8, it appears that indirect genetic effects on overall ADHD trait magnitude may be more difficult to estimate with precision than subdomain-specific effects.

## Background

Attention-deficit hyperactivity disorder (ADHD) is a neurodevelopmental condition comprising developmentally inappropriate and impairing inattention, hyperactivity and/or impulsivity (Faraone et al. 2021). Clinically meaningful trait differences typically have a childhood onset and persist at clinically severe or subthreshold levels into at least young adulthood (Nigg et al. 2020). A wide range of twin studies have shown that ADHD is highly heritable, with 60–90% of variance in ADHD being explained by genetic factors and 10–40% explained by environmental factors (Eilertsen et al. 2019; Sciberras et al. 2017). However, genetic and environmental influences do not operate independently, and estimates of genetic and environmental effects can be inaccurate if the methods used to obtain them do not adequately account for gene-environment interplay (Rijsdijk and Sham 2002). While genetic research is often implicitly focused on understanding direct genetic effects—the direct influence of an individual’s genotype on their own phenotype—several studies have emphasized that phenotypes are also subject to the indirect genetic effects of other peoples’ genotypes via the environment (Kong et al. 2018; Young et al. 2018). Where these environmentally mediated effects occur within nuclear families, they are known as genetic nurture effects. For example, a child’s household environment is in part shaped by the genetically driven behaviors of parents and siblings, which can exert genetic nurture effects on children’s behavior, physical health, and other developmental processes (Kong et al. 2018). Genetic nurture effects can correlate with direct genetic effects (a form of passive gene-environment correlation; rGE), and thus potentially bias estimates of direct genetic effects. Genetic nurture effects are also interesting in their own right as they can provide a broad indication of the impact of family members on children’s development.

Recently, several approaches have been used to estimate genetic nurture effects on a focal individual’s phenotype using genotype data from their family members. One approach uses polygenic scores (PGSs), individualized scores of genetic liability for a phenotype constructed using the weighted effect sizes of single-nucleotide polymorphisms (SNPs) found to account for phenotypic variance in genome-wide association studies (GWAS) (Rosenberg et al. 2019). When individual PGSs are constructed for children and their parents, and their effects modelled jointly, genetic nurture effects can be inferred when parental PGSs predict child phenotypes after accounting for the effects of child genotype (Balbona et al. 2021). Taking this approach, a recent study by Pingault et al. (2022) found that mothers’ PGSs for neuroticism (but not ADHD) predicted children’s ADHD traits over and above their own PGS for ADHD. This provides evidence for maternal genetic nurture effects on children’s traits, but that these were driven by mothers’ genetic risk for neuroticism rather than ADHD itself. Another study by de Zeeuw et al. (2020) found that children’s ADHD traits were associated with their own PGSs for adult ADHD and educational attainment, but not with PGSs constructed from the 50% of non-transmitted SNPs from each parent. This was interpreted as indicating a lack of genetic nurture effects on child ADHD traits.

Genetic nurture effects can also be estimated using extensions of SNP heritability methods such as genome-wide complex trait analysis (GCTA), a method of estimating the portion of phenotypic variance accounted for by variance in common SNPs (Yang et al. 2011). While conventional GCTA aims to estimate direct genetic effects, it does not account for the fact that the phenotypic variance accounted for by any given SNP in an individual could be due in part to genetic nurture effects, i.e., by affecting the behavior of family members who share the same SNP (Young et al. 2018). For example, SNPs exerting direct genetic effects on children’s ADHD traits may, in parents, affect the harshness or consistency of their parenting, which may exert additional effects on children’s ADHD traits (Johnston et al. 2012; Park et al. 2017). These effects can be quantified by jointly modelling the indirect effects of parent genotypes alongside the direct genetic effects of the child genotype. For example, maternal GCTA (Eaves et al. 2014) and trio-GCTA (Eilertsen et al. 2021) can estimate child, maternal, and paternal genetic effects separately, while Relatedness Disequilibrium Regression (RDR) estimates child genetic effects alongside a combined parental genetic effect (Young et al. 2018). Notably, for RDR and trio-GCTA, the availability of genetic data from both parents allows for the unbiased estimation of SNP heritability (i.e., direct child genetic effects) while accounting for genetic nurture effects and any effects of population stratification and assortative mating.

A key strength of trio-GCTA and related methods is that they capture the cumulative contribution of all heritable characteristics of family members on a child phenotype up to the time of measurement, without needing to measure specific characteristics (Cheesman et al. 2020; Eilertsen et al. 2021). This can complement and inform existing research efforts to assess the importance of family-level influences on child development. For example, observational researchers have sought to establish the effects of parenting on children’s ADHD traits, typically relying on retrospective parent reports of engaging in specific behaviors over a certain time period, which are hindered by recall and rater biases (Claussen et al. 2022; Deault 2010; Putnick 2019). To date, a small number of genetically sensitive studies have demonstrated that certain parental risk factors, such as child-directed hostility and abuse/neglect, predict or are associated with higher child ADHD traits (Harold et al. 2013; Stern et al. 2018). However, the relative importance of specific parent behaviors in the development of ADHD is difficult to discern among the wide range of inter-correlated behaviors and parent-child interactions that occur across development. While it is virtually impossible to simultaneously measure all child exposures that may impact ADHD, trio-GCTA provides an opportunity to estimate the cumulative impact of all heritable parent behaviors and characteristics up to a given age.

Recently, Eilertsen et al. (2022) used trio-GCTA to assess direct and indirect genetic effects on several child externalizing phenotypes in 9,675 trios of children and parents from the Norwegian Mother, Father and Child Cohort Study (MoBa) birth cohort. They reported substantial indirect genetic effects of parents’ combined genotype on children’s conduct, inattention, and hyperactivity traits (but not oppositional defiant traits) at age 8, alongside the larger direct genetic effects of children’s own genotype on all measures. While some portion of these indirect effects could be due to assortative mating and residual population stratification, these results provide evidence for genetic nurture effects on several externalizing phenotypes in mid-childhood, including the two main trait domains of ADHD.

Trio-GCTA could also help to better understand the relative importance of genetic and environmental effects on ADHD traits across development. Twin research on ADHD has shown a slight increase in the heritability of ADHD from early to mid-childhood, with non-shared environmental effects explaining the remaining variance (Kuntsi et al. 2005). Genetic effects have also been shown to account for most of the stability of ADHD traits across childhood, adolescence, and early adulthood, although newly arising genetic and non-shared environmental factors do account for additional variance at later ages (Chang et al. 2013; Kuntsi et al. 2005). As discussed previously, however, estimates of genetic and environmental effects may be biased by passive rGE including unmeasured genetic nurture effects. Trio-GCTA can shed light on age-related etiological differences in ADHD by quantifying the relative importance of direct and indirect genetic effects at different ages. However, to date, no studies have explored developmental change in ADHD using methods that can distinguish direct and indirect genetic effects.

Here, we set out to assess direct and indirect genetic effects on children’s ADHD traits using trios of children and their parents from the MoBa cohort. Whereas past work has examined direct and indirect genetic effects on children’s hyperactivity-impulsivity and inattention subdomains at specific ages, we focused on developmental change in direct and indirect genetic effects on total ADHD traits across two developmental stages. Specifically, we were interested in whether there were genetic nurture effects on children’s ADHD traits at age 5, and whether there were age-related changes in the relative importance of direct and indirect effects on ADHD traits by age 8.

## Methods

**Table 1:** Descriptive statistics for age 5 and age 8 phenotypic subsamples

Measure	N*	Mean	SD	Min.	Max.	Skew	Kurtosis	SE
Age 5								
Parity (no. previous births)	15,004	0.67	0.83	0	4	1.19	1.16	0.01
Maternal age at childbirth	15,004	30.53	4.33	17	45	0.10	−0.10	0.04
Year of childbirth	15,004	2006.10	1.48	2004	2009	0.07	−1.05	0.01
Child ADHD Age 5 (CPRS-R)	15,004	1.36	0.37	1	4	1.90	5.77	0.00
Age 8								
Parity (no. previous births)	15,498	0.72	0.85	0	4	1.10	0.90	0.01
Maternal age at childbirth	15,498	30.58	4.29	17	45	0.05	−0.06	0.03
Year of childbirth	15,498	2005.50	1.85	2002	2009	−0.05	−0.97	0.01
Child ADHD Age 8 (RS-DBD)	15,498	1.46	0.38	1	3.89	1.74	4.42	0.00

*Analytic subsamples of 12,374 (age 5) and 12,714 (age 8) were derived for final analyses after excluding non-parent-child pairs with *r*_*SNP*_ > 0.10.

*ADHD* Attention-deficit hyperactivity disorder, *CPRS-R * Conners Parent Rating Scale-Revised Short Form, *RS-DBD * Parent/Teacher Rating Scale for Disruptive Behavior.

### Sample

MoBa is a prospective population-based birth registry cohort of 114,500 children, 95,200 birth mothers and 75,200 birth fathers in Norway (Magnus et al. 2016). Data collection for MoBa covered pregnancies across all of Norway from 1999 to 2008, with 40.6% of eligible pregnant women consenting to participate in the study. The current study is based on version 12 of the quality-assured MoBa data files released for research in January 2019. Blood samples were obtained from both parents during pregnancy and from mothers and children (umbilical cord) at birth. Quality-controlled genotyping array data was recently generated for the full 207,569 unique MoBa participants as part of the MoBaPsychGen pipeline (Corfield et al. 2022). Therein, phasing and imputation were performed with IMPUTE4.1.2_r300.3, using the publicly available Haplotype Reference Consortium release 1.1 panel as a reference. To identify a sub-population of European-associated ancestry, principal component analysis (PCA) was performed with 1000 Genomes phase 1 after LD-pruning. During post-imputation quality control, the following thresholds were used for SNP removal: imputation quality (INFO) score ≤ 0.8; MAF < 1%; call rate < 95%. This produced a final sample of 6,981,748 SNPs across 207,569 unique individuals (Corfield et al. 2022). After linking with phenotypic data, we derived phenotypic subsamples of 15,004 genotyped trios where child ADHD data were available at age 5, and 15,498 trios where data were available at age 8. These subsamples were largely overlapping, with 10,675 trios with child ADHD data available at both ages. There was a roughly equal number of boys and girls at both ages (50.5% and 50.9% male at ages 5 and 8 respectively). Descriptive statistics for the sample are displayed in Table 1.

### Measures

Mothers reported children’s ADHD traits at age 5 years using the Conners Parent Rating Scale-Revised (CPRS-R) Short Form. The CPRS-R is a well-validated measure of parent-reported child ADHD traits, constructed from items found to best discriminate clinically diagnosed children from community controls matched for age, sex, and ethnicity (Conners et al. 1998). It includes 12 items assessing children’s inattentive, impulsive, and hyperactive traits. Mothers reported children’s ADHD traits at age 8 years using the Revised Short Form of the Parent/Teacher Rating Scale for Disruptive Behavior (RS-DBD). The RS-DBD is a measure of parent-reported child ADHD traits which includes 18 items assessing children’s inattentive, impulsive and hyperactive traits (Silva et al. 2005). Scores on the CPRS-R and the RS-DBD had excellent internal consistency (Cronbach’s *α* = 0.88; 0.91). For the overlapping set of 10,675 trios where child ADHD traits measures were available at both ages 5 and 8, mothers’ ratings of children’s ADHD traits were moderately correlated (Pearson’s *r* =.55).

### Analyses

We controlled for the effects of maternal age, parity (mother’s number of births), and child sex by regressing their effects out of child ADHD measures. In addition, to minimize the contribution of population stratification, 40 principal components (the first 20 from each parent) of genetic ancestry from the MoBaPsychGen dataset (Corfield et al. 2022) were regressed out of child ADHD measures. Genotype batch groups were also regressed out to control for batch effects. We applied Box-Cox transformations (Osborne 2010) on the resulting standardized residuals to reduce non-normality.

Genomic relatedness matrices (GRMs) were computed for all individuals (Yang et al. 2011), based on a subset of ~ 1 million high-quality SNPs that were genotyped at least 5 times across the six batches, and/or imputed with INFO quality scores > 0.98. Pairs of individuals whose SNP-based genetic correlations exceeded 0.10 (who were not parent-child pairs) were excluded from analyses, to minimize the potential for confounding by passive rGE (e.g. whereby genetic relatedness correlates with environmental similarity). This produced analytic subsamples of 12,374 trios at age 5 and 12,714 trios at age 8. Trio-GCTA analyses were then conducted using the VCModels package (Eilertsen et al. 2021) in Julia 1.5.3. (Bezanson et al. 2017). Full models simultaneously estimated the direct effects of children’s genotype and the indirect effects of maternal and paternal genotypes. Covariances between these effects were also modelled, while all variance not accounted for by SNP variation was captured in a residual term (Eilertsen et al. 2021). Several nested models were tested, with Combined models constraining maternal and paternal effects to be equal, providing a combined parental indirect genetic effect (equivalent to RDR), Direct models estimating only child direct genetic effects (equivalent to standard GCTA), and Null models constraining all genetic effects to zero. Likelihood ratio tests were used to assess whether constraining parameters led to a significant worsening in model fit at an α threshold of 0.05. To adjust for multiple testing, false discovery rate (FDR) corrected p-values adjusting for three tests per outcome were computed using the Benjamini-Hochberg procedure (Benjamini and Hochberg 1995).

**Table 2:** Standardized parameter estimates (SEs) and model fit statistics from age 5 and age 8 analyses, comparing full models estimating all genetic effects separately, combined parental effects models constraining maternal and paternal genetic effects to be equal, direct models constraining parental genetic effects to zero, and null models constraining all genetic effects to zero

Effects on child ADHD traits at 5 years												
Model	$\:{\sigma\:}_{o}^{2}$	$\:{\sigma\:}_{m}^{2}$	$\:{\sigma\:}_{p}^{2}$	$\:{\sigma\:}_{om}$	$\:{\sigma\:}_{op}$	$\:{\sigma\:}_{mp}$	$\sigma \:_{ \in }^{2}$	*−2LL*	*AIC*	*df*	p-value	Adj. p-value
Full	0.018 (0.020)	0.047 (0.026)	0.000 (0.002)	0.029 (0.012)	− 0.001 (0.010)	− 0.002 (0.016)	0.907 (0.029)	35,078.89	35,094.89	7		
Combined	0.013 (0.020)	0.016 (0.014)*		0.010 (0.006)*		-	0.933 (0.029)	35,084.90	35,094.90	4	0.111	0.111
Direct	0.045 (0.027)	-	-	-	-	-	0.955 (0.029)	35,088.27	35,094.27	2	0.095	0.111
Null	-	-	-	-	-	-	1 (0.013)	35,091.15	35,095.15	1	0.056	0.111

Effects on child ADHD traits at 8 years												
Model	$\:{\sigma\:}_{o}^{2}$	$\:{\sigma\:}_{m}^{2}$	$\:{\sigma\:}_{p}^{2}$	$\:{\sigma\:}_{om}$	$\:{\sigma\:}_{op}$	$\:{\sigma\:}_{mp}$	$\sigma \:_{ \in }^{2}$	*−2LL*	*AIC*	*df*	p-value	Adj. p-value
Full	0.083 (0.047)	0.082 (0.039)	0.003 (0.007)	− 0.023 (0.035)	− 0.009 (0.022)	0.014 (0.021)	0.864 (0.038)	36,061.26	36,077.26	7		
Combined	0.063 (0.053)	0.006 (0.026)*		0.005 (0.033)*		-	0.915 (0.038)	36,067.17	36,077.17	4	0.116	0.174
Direct	0.079 (0.026)	-	-	-	-	-	0.921 (0.028)	36,067.61	36,073.61	2	0.274	0.274
Null	-	-	-	-	-	-	1 (0.013)	36,077.28	36,081.28	1	0.014	0.042

FDR-corrected p-values for likelihood ratio tests are provided in the final column alongside standard p-values.

*Combined models jointly estimate parental genetic effects ($\:{\sigma\:}_{pp}^{2}$) and correlations between child and parental genetic effects ($\:{\sigma\:}_{opp}$).

*ADHD* Attention-deficit hyperactivity disorder, $\:{\sigma\:}_{o}^{2}$, $\:{\sigma\:}_{m}^{2}$, $\:{\sigma\:}_{p}^{2}$ Child, maternal and paternal genetic effects, $\:{\sigma\:}_{om}$, $\:{\sigma\:}_{op}$, $\:{\sigma\:}_{mp}$ Correlations between child, maternal and paternal genetic effects, $\sigma \:_{ \in }^{2}$ Variance not explained by genetic effects.

## Results

Standardized parameter estimates with standard errors and fit statistics for all models are displayed in Table 2. In the age 5 Full model, child direct genetic effects explained 1.8% of variance in child ADHD traits while maternal indirect genetic effects explained 4.7% of variance. Paternal indirect genetic effects explained no variance. While the Direct model had the best fit as indicated by AIC, the Null model was not significantly worse fitting compared to the Full model nor the Combined or Direct models, indicating a lack of both direct and indirect genetic effects on ADHD traits at age 5.

In the age 8 Full model, child direct genetic effects explained 8.3% of variance in children’s ADHD traits while maternal and paternal indirect genetic effects explained 8.2% and 0.3% of variance, respectively. The Direct model demonstrated the best fit, with a lower AIC than the Full, Combined, and Null models, and a comparable estimate of direct genetic effects to the Full model. The Null model was significantly worse fitting than the Full, Combined, and Direct models. This suggests that while child direct genetic effects were significant and consistently detected, there was a lack of power to distinguish direct and indirect genetic effects when these were modelled jointly.

## Discussion

We set out to assess whether maternal and paternal genotypes exerted indirect effects on children’s ADHD traits over and above the direct effects of children’s own genotype. We also assessed the potential for developmental change in the magnitude and relative proportions of direct and indirect genetic effects from early to mid-childhood.

Full models indicated a shift in the relative magnitude of direct and indirect genetic effects, with predominantly maternal indirect genetic effects and only minor direct genetic effects on ADHD traits at age 5, followed by a later pattern of substantial and roughly equal direct genetic effects and maternal indirect genetic effects on ADHD traits at age 8. However, only the Direct model at age 8 performed significantly better than the Null model as per nested model comparisons, with likelihood ratio tests ultimately indicating an absence of indirect genetic effects on ADHD traits at both ages, as well as an absence of direct genetic effects at age 5. Furthermore, in all but the Direct model at age 8, the relative importance of specific effects could not be interpreted with confidence due to large standard errors for most estimates, evidencing a lack of statistical power to estimate both direct and indirect genetic effects. Previous findings by Eilertsen et al. (2022) were also subject to issues with statistical power, while using partially overlapping measures of hyperactivity and inattention at age 8 with a smaller analytical sample of 9,675 trios (a subsample of the children in the current study). However, they did find evidence for indirect genetic effects on several externalizing phenotypes. The fact that we did not find any clear evidence for indirect genetic effects at age 5 nor age 8, despite a ~ 25% increase in sample size, is surprising.

The most stringent interpretation of our findings is that, especially given that indirect genetic effects have been estimated with confidence in previous studies, this not being the case in our analyses suggests that there are few if any such effects meaningfully operating on child ADHD traits at ages 5 and 8. This is not completely at odds with past findings. Previously, de Zeeuw et al. (2020) and Pingault et al. (2021) found no evidence for genetic nurture effects of parents’ ADHD PGSs on children’s ADHD traits, despite the latter study using the same age 8 child ADHD measure as our study, also in a subset of the MoBa sample. The key distinction of PGS approaches is that their inferences of genetic nurture rely on parents’ PGSs for specific phenotypes, with SNPs weighted by the variance they explain in that specific parental phenotype (itself typically measured at one point in time). Interestingly, Pingault et al. (2021) did find that child ADHD traits were predicted by mothers’ PGSs for neuroticism, suggesting that while there are genetic nurture effects on children’s ADHD traits, these do not appear to be caused by SNPs that raise parents’ own risk for ADHD. Estimates of indirect genetic effects from both trio-GCTA and PGS approaches can also include residual population stratification effects, i.e., the effects of environmental factors that are correlated with SNP variation in parents but are not caused by parent characteristics or behaviors. These could include social or other environmental effects associated with specific geographical regions or socioeconomic conditions.

In contrast to PGS approaches, trio-GCTA captures the indirect effects of all genotyped parental SNPs on a child phenotype, maximizing the chance of observing indirect genetic effects. However, these effects are not always detected, and whether they can be estimated precisely depends not only on sample size. For instance, while the trio-GCTA by Eilertsen et al. (2022) found evidence for substantial genetic nurture effects on three of the four externalizing phenotypes they analyzed (inattention, hyperactivity, and conduct disorder traits, but not oppositional defiant disorder traits), there were notable differences across outcomes even between ADHD subdomains. Their likelihood ratio tests favored a Combined model with a joint parental indirect genetic effect on hyperactivity at age 8, but a Direct model with no indirect genetic effects on inattention at age 8. Conversely, standard errors were substantially larger for estimates of both direct and indirect genetic effects on hyperactivity compared to the equivalent estimates for inattention, despite equal model complexity and nearly equal sample sizes across outcomes. This suggests that the choice of examined outcomes can have consequences both for the precision of specific parameter estimates and for overall model fit. It could be that our choice of examining total ADHD trait scores as outcomes rather than subdomains obscured subdomain-specific direct and/or indirect genetic effects. As the focus of our study was on developmental change in genetic nurture effects on overall ADHD trait magnitude rather than on specific subdomains, one interpretation of our findings could be that indirect genetic effects do not appear to operate as strongly on total ADHD trait magnitude as they do on specific subdomains like hyperactivity.

The lack of direct genetic effects on child ADHD traits at age 5 was also an unexpected finding, appearing to suggest that while children’s common SNP variation exerted a substantial effect on their ADHD traits in mid-childhood, it had relatively little effect on these traits in early childhood. This is surprising given the high heritability of ADHD, particularly at younger ages (Faraone et al. 2021). While the difference seen between ages 5 and 8 could be partly due to the different ADHD measures available at these ages, a lack of direct genetic effects in early childhood could also be interpreted as broadly consistent with findings that, across a broad range of emotional and behavioral phenotypes, children’s individual genetic propensities account for more phenotypic variance as they grow older, while the relative role of common environmental effects declines (Hannigan et al. 2017). Differences in age at onset of ADHD could also mean a portion of the children in our sample who go on to manifest clinically meaningful ADHD traits by age 8 do not differ substantially from their peers at age 5. However, a recent trio-GCTA study by Hegemann et al. (2025) suggests that these explanations may not fully account for the lack of direct genetic effects in our study. The study focused on a range of neurodevelopmental outcomes in MoBa children at age 3, with the lack of attrition at this age allowing analyses to benefit from a sample of ~ 24,500 trios, twice as large as that of the current study. The authors found evidence for direct genetic effects on all but one of the six neurodevelopmental outcomes analyzed at age 3, including hyperactivity and inattention. This finding is notable given that the onset of ADHD traits is after this age in most children (Rocco et al. 2021), suggesting that even early variation in hyperactivity and inattention is subject to the genetic influence of common SNPs.

The findings reported by Hegemann et al. (2025) can also shed light on other questions regarding our and previous trio-GCTA analyses, in particular whether the inability to precisely estimate indirect genetic effects on ADHD traits primarily reflects a lack of statistical power that could simply be addressed with larger samples or different design choices, as opposed to reflecting a true lack of indirect genetic effects on ADHD traits at ages 5 and/or 8. Despite focusing on an age where notable ADHD traits will not have manifested for most children, hyperactivity and inattention were the two outcomes for which they reported the clearest evidence of indirect genetic effects. Interestingly, maternal indirect genetic effects on both hyperactivity and inattention were found to be larger than direct genetic effects, particularly for hyperactivity, with maternal indirect genetic effects explaining 8.5% of variance while direct genetic effects explained only 1.3% of variance (with a large standard error of 2%). Along with the lack of paternal indirect genetic effects, this pattern of findings bears a close resemblance to our age 5 Full model findings of predominantly maternal indirect genetic effects on total ADHD trait scores at age 5. Furthermore, the more equal pattern of direct and indirect maternal genetic effects on inattention Hegemann et al. (2025) observe at age 3 (explaining 4.8% and 6.7% of variance respectively), accompanied by a lack of paternal indirect genetic effects, resembles our Full model results at age 8, where direct and maternal indirect genetic effects both explained larger and roughly equal portions of variance at around 8%, but the estimate of the maternal effect was estimated with substantially higher precision. While these findings at age 3 do not directly bear on the validity of our findings at ages 5 and 8, they do demonstrate that the pattern of direct and indirect genetic effects on ADHD traits we observe in our Full models resembles a pattern estimated with much higher confidence in a larger sample of the children in our study at an earlier age. Similarities are evident in estimates of specific indirect genetic effects as well as in the unexpected lack of direct genetic effects on ADHD traits in early childhood. Therefore, while it may not be possible to draw confident inferences regarding indirect genetic effects from our findings, we would argue that it is inadvisable to dismiss them outright.

With the above in mind, we cautiously consider the possibility that our inability to distinguish indirect genetic effects at ages 5 and 8 with confidence may have been driven primarily by our use of total ADHD trait scores as outcomes, such that subdomain-level effects observed in previous studies at ages 3 and 8 were obscured. The pattern of developmental change in direct and indirect genetic effects we observed between ages 5 and 8 could reflect differences in ADHD measures used within our study (both of which differed from the Child Behavior Checklist measure at age 3), but could also reflect a decline in hyperactivity traits in children beyond early childhood, such that our total ADHD score more strongly indexed variation in children’s inattention by age 8. If so, this could explain the resemblance between our findings of predominantly maternal indirect genetic effects on ADHD traits at age 5 and those reported for hyperactivity by Hegemann et al. (2025), and the resemblance between our findings of more equal direct and maternal indirect genetic effects on ADHD traits at age 8 and those reported in their analysis of inattention. Finally, we would caution against overinterpreting the finding of direct genetic effects on ADHD traits at age 8 based only on model comparison tests, given that while estimates of these direct genetic effects were nearly identical between Full and Direct models, the estimate in the Full model had a much larger standard error. Indeed, in the Direct model the estimate of maternal indirect genetic effects was roughly equal but had a substantially smaller standard error. It therefore appears plausible that the estimate of direct genetic effects in the Direct model, while being the most precise, may capture both direct genetic effects and the indirect genetic effects of child SNPs inherited with mothers on ADHD traits at age 8, which were not modelled separately in the Direct model.

### Strengths and Limitations

We used a large, representative sample of genotyped child, mother and father trios combined with a novel methodology to assess the indirect effects of maternal and paternal genotypes on children’s ADHD traits in early and mid-childhood, over and above the direct effects of children’s own genotype. This allowed us to assess the cumulative effect of all parental behaviors, characteristics, and associated environmental exposures tagged by parental SNPs on children’s ADHD traits up to ages 5 and 8. However, there were several limitations. First, a lack of statistical power was evidenced by the inability to distinguish best-fitting models and by notably large standard errors for most of our parameter estimates. Larger samples may be necessary to detect the full range of direct and indirect genetic effects on child ADHD traits and interpret findings with confidence. Second, examining total ADHD trait scores may have obscured subdomain-specific direct and indirect genetic effects. Third, children’s ADHD traits were reported by mothers, so ratings could have been subject to rater biases and mothers’ perceptions based largely on their own interactions with children. While father, teacher, and clinician ratings are difficult to collect for the large samples needed for trio-GCTA, medical records with clinically ascertained diagnoses of ADHD could be used in future studies. Fourth, our sample was limited to genotyped trios of European ancestry, which may limit generalizability of findings to non-European populations.

## Conclusions

We observed a pattern of developmental change in the relative magnitude of direct and indirect genetic effects on child ADHD traits between early and mid-childhood, with maternal indirect genetic effects being the main source of genetically driven trait variation in early childhood, followed by a roughly equal pattern of child direct genetic effects and maternal indirect genetic effects in mid-childhood. While these findings are not wholly inconsistent with past work, our model fit comparisons favored models with no indirect genetic effects on child ADHD traits in either early or mid-childhood, and direct genetic effects only in mid-childhood. While our focus was on genetic nurture effects on overall ADHD traits rather than on specific subdomains, analyzing total ADHD scores may have obscured subdomain-specific effects as reported in prior trio-GCTA studies. Therefore, while the pattern of results we observed may be informative, our findings as a whole appear to indicate a relative lack of indirect genetic effects on total ADHD trait magnitude as opposed to effects on specific subdomains.

Declarations.

## Data Availability

No new data were collected or analysed as part of the current study. While access to MoBa phenotypic and genotype data is restricted, the MoBa dataset and its relevant overviews and data dictionaries are available at https://www.fhi.no/en/studies/moba/.
